# Citalopram-induced sleep bruxism in a breastfed infant: A case report

**DOI:** 10.3389/fpsyt.2023.1051346

**Published:** 2023-02-02

**Authors:** Farzad Akbarzadeh, Ghazal Behravan, Farzaneh Modaresi, Mahboubeh Eslamzadeh

**Affiliations:** ^1^Psychiatry and Behavioral Sciences Research Center, Faculty of Medicine, Mashhad University of Medical Sciences, Mashhad, Iran; ^2^Department of Psychiatry, Fasa University of Medical Sciences, Fasa, Iran

**Keywords:** bruxism, citalopram, selective serotonin reuptake inhibitors (SSRIs), breastfeeding, side effect, antidepressant, case report

## Abstract

Bruxism associated with antidepressant use is an under-recognized phenomenon. The use of citalopram has gained wide acceptance in the treatment of depression and anxiety disorders; however, the consumption of this medication during lactation and pregnancy has not been carefully characterized. There are limited studies about its side effects in the breastfeeding period. Here, we report a rare case of citalopram-induced sleep bruxism in a 9-month-old female breastfed infant whose mother used SSRI agent citaloporm for her anxiety disorder. Within 2 weeks of initiating her citalopram treatment, with a starting dose of 10 mg/day, the patient reported sleep bruxism in her infant. Thorough examinations of the infant were performed and no abnormal finding was reported. After ruling out other possible causes, the new-onset bruxism symptoms were attributed to the mother’s recent use of citalopram, which was discontinued thereafter. The infant’s symptoms of bruxism disappeared following the discontinuation of the medication by her mother. These findings and similar reports could draw more attention to bruxism or other possible symptoms in breastfed infants of mothers consuming psychotropic medications.

## Introduction

Selective serotonin reuptake inhibitors (SSRIs) are the most prevalent drugs utilized for the treatment of major depressive disorder. SSRIs work by targeting the serotonin transporter (SERT),^1^ which leads to higher extracellular levels of serotonin; this is considered the basis of their mechanism of action, although other mechanisms have been proposed (1). SSRIs are generally safe and well tolerated. However, certain side effects have been reported to be associated with the use of SSRIs, including: sexual dysfunction, dry mouth, bleeding, weight gain, and gastrointestinal symptoms (2). They also have some side effects on the central nervous system, including akathisia, tremor, dystonia, and bruxism (3, 4).Antidepressant-associated bruxism may occur in pediatric and adult patients, most commonly among female patients (5). Both short-term and long-term use of antidepressants have been associated with bruxism. The most commonly reported offending agents are fluoxetine, sertraline, and venlafaxine. Symptoms may begin within 3–4 weeks of medication initiation and may resolve within 3–4 weeks of drug discontinuation, with the addition of buspirone, or by substitution with another pharmacologic agent. The incidence of this phenomenon is unknown (5).

“Bruxism” is an umbrella term under which fall various motor activities of the jaw muscles, such as grinding and clenching of the teeth as well as thrusting of the mandible (6). According to Lobbezoo et al., sleep bruxism and awake bruxism have been defined as “a masticatory muscle activity during sleep, characterised as rhythmic or non-rhythmic in otherwise healthy individuals” and “a masticatory muscle activity during wakefulness characterised by repetitive or sustained tooth contact and/or bracing or thrusting of the mandible in otherwise healthy individuals” respectively. It should also be noted that in both definitions, bruxism is not considered a movement disorder or sleep disorder (7).

Bruxism, characterized by teeth clenching or grinding, is considered an involuntary nonfunctional activity of the masticatory system. It might happen during sleep or while awake, consciously or unconsciously, and is not classified as a disorder, but as a behavior. This habit is common during childhood. Its prevalence in children ranges from 13 to 49% (8) and may have negative consequences on the stomatognathic system (9). SSRI’s, including citalopram, have been shown to potentially induce sleep and/or awake bruxism (10).

Intergenerational effects, whether through medication or other means, have increasingly become of interest, especially in the field of psychiatry (11). We here, would like to report a case of citalopram-associated sleep bruxism in a female breastfed infant, which resolved by discontinuation of her mother’s medication.

## Case presentation

A 33-year-old Persian female patient referred to Ibn-Sina outpatient psychiatry clinic in Mashhad, Iran, in June 2019, with a mixed anxiety depressive disorder during her breastfeeding period. She had a history of anxiety prior to her pregnancy, and was under treatment with sertraline. She had previously shown symptoms of agitation and become irritable with her sertraline treatment, and had no tendency to continue using this medication. The patient was unemployed; described her family’s economic status as “stable” and mentioned she had “strong emotional support” from her spouse. Her past surgical history included only a recent Cesarean section. She was breastfeeding her then, 9-month-old infant. Pregnancy seemed to have been a significant psychosocial stressor for her. The patient presented to our general adult psychiatry outpatient clinic with a 3-month history of mood symptoms; including low mood, anhedonia, insomnia, anxiety, loss of energy, and decreased appetite. Symptoms had significantly worsened over the 1-month period before she referred to us.

She had no suicidal or homicidal thoughts, intent, or plans as well as no history of suicide attempts or self-harm. The patient also had no history of psychotic symptoms or manic episodes. She had never smoked, nor used alcohol, cannabis, or any illicit substances. There were no significant findings in her physical examination. Her electrocardiography (EKG), including her corrected QT interval (QTc) was normal. Considering the patient’s past history of sertraline-associated agitation, a 10-mg daily starting dose of citalopram was prescribed for her. Her symptoms began to decline 10 days after her consumption of citalopram was initiated. However, 2 weeks after the initiation of her treatment, she reported symptoms of bruxism in her infant. The infant was a term 9-month-old female, delivered through Cesarean section after an uneventful pregnancy, with no history of physical disorders, illnesses, or hospital admissions and no use of medications except supplements. The infant’s diet consisted of her mother’s breast milk five to six times per day, as well as standard supplementary nutrients given for 9-month-old infants consisting of such as fruits, vegetables, meat, and biscuits. She was not being fed with milk powder or any other formula and was not consuming any medications. The patient reported that her infant had sporadic, pulsatile, and momentary movements in her jaws, which usually began with movements of the head, especially during sleep. Furthermore, the mother mentioned her child had a habit of biting and clenching her teeth while awake.

### Clinical findings

A thorough physical examination, including extra-oral and intra-oral assessment of the child by a pediatrician showed no abnormalities. Another intraoral examination was performed by a pediatric dentist who reported appropriate teeth growth and development in the child. The dentist reported no pathologic findings. The infant had no salivary drooling and was able to swallow efficiently with no evident cranial nerve dysfunction. However, bruxism was observed by the dentist during the examination.

### Diagnostic assessment

After somatic and organic problems were ruled out, the infant’s symptoms of bruxism were attributed to the excretion of citalopram in the mother’s milk.

### Therapeutic intervention

Citalopram, which was hypothesized as the main cause of the child’s symptoms of bruxism, was discontinued, with bruxism symptoms resolving 72 h thereafter.

### Follow-up and outcomes

The mother and her child were followed for 2 years. The infant’s symptoms of bruxism stopped after her mother’s use of citalopram was discontinued. She no longer had teeth clenching or grinding while awake or during sleep. The mother resumed to breastfeeding right away after the infant’s symptoms disappeared with no further consequences and was referred to a psychotherapist for cognitive behavioral therapy (CBT) with improvements in both her depressive and anxiety symptoms.

## Discussion

In this paper, we report sleep bruxism in a breastfed 9-month-old infant whose mother was under treatment with citalopram. The point of this case was to bring attention to the excretion of medications like SSRIs into breastmilk. Considering the onset of bruxism in the infant shortly after the initiation of her mother’s citalopram treatment and the cessation of this symptom following the discontinuation of the medicine, the infant’s symptoms of bruxism were attributed to her mother’s use of citalopram after ruling out other physical or organical causes. This is unique, as there are no similar studies in the current literature.

There is transfer of SSRI’s including citalopram and escitalopram to human breast milk. A study by Pogliani et al. reported that all lactating women treated with fluoxetine or citalopram had relative infant doses (RID’s) exceeding 10% (12). Previous studies have reported weight loss and drowsiness in breastfed infants of mothers who used citalopram (13). It should also be noted that the individual genetical characteristics of the mother and/or infant may also play a role in how their body metabolizes and responds to a medication. It has been reported that the polymorphism of CYP2C19 plays a crucial role in the N-demethylation of citalopram. As a result, poor metabolizers and extensive metabolizers of CYP2C19 show a significant difference in the disposition of citalopram *in vivo* (14). In this patient, the symptoms regressed merely by cessation of the medication. This was possibly due to the low level of the drug in the infant’s serum.

A review paper by Wallem et al. had also concluded that there is an “apparent” relationship between treatment with SSRI’s and the development of bruxism (15). Another systematic review by Melo et al. had found an association between the development of sleep bruxism in children and the use of psychotropic medications such as duloxetine, paroxetine, venlafaxine, and methylphenidate. However, they found no significant correlation between symptoms of bruxism and the utilization of medications such as citalopram, escitalopram, fluoxetine, and mirtazapine (16).

It is also noteworthy to take into consideration the possible effects of intra-uterine exposure to SSRI’s on fetus brain development (17), although our patient was not using any antidepressant medication during her pregnancy. It is also of importance to note the fact that many factors in the early home environment can impact brain development in infants and children (18). According to Orsolini et al., paroxetine and sertraline have better neonatal safety profiles during breastfeeding compared to other SSRI medications (19).

Several therapeutic modalities have been suggested, but there is no consensus about which is the most efficient. Some studies have reported hydroxyzine to be an efficient treatment for sleep bruxism in children (20). Occlusal splints, orthodontic, physical, and psychological interventions have also shown to be effective in reducing bruxism (21).

Generally, symptoms tend to appear within 3–4 weeks of the initiation of antidepressant treatment or of undergoing dose titration. Symptom resolution may be achieved the addition of serotonin 1A (5HT1A) partial agonists (buspirone, tandospirone), by dose reduction, by medicine cessation, or by the addition of other pharmacologic agents including tricyclic antidepressants (amitriptyline), antipsychotics (aripiprazole, chlorpromazine), opipramol, norepinephrine-dopamine reuptake inhibitors (bupropion), or serotonin 2A/2C antagonist and reuptake inhibitors (trazodone) (22). Studies have also shown that clonidine can effectively reduce symptoms of sleep bruxism (23). Symptoms may also resolve over time without pharmacological intervention. The use of low-dose quetiapine has also been reported to be effective in the treatment of mandibular dystonia and SSRI-induced bruxism (24). The addition of buspirone has been reported to be successful in alleviating symptoms of bruxism in several cases. Buspirone, a weak 5-HT2C receptor antagonist, can potentially compete with serotonin to bind with 5-HT2C receptor, which can lead to improvement of bruxism (25).

This observation along with considering medications used for bruxism treatment can be instructive in both understanding the underlying pathophysiological mechanisms of antidepressant-associated bruxism, as well as in providing a foundation for treatment recommendations.

## Limitations and suggestions for future studies

One of the limitations of this study is that since it is a case report, direct causal relationships cannot be determined. We should also take into account that as previously cited, given how common bruxism is in children, the infant’s symptoms of bruxism may have been caused by other factors. In this patient, 2 weeks after the consumption of the drug (and thus, its excretion into the mother’s breastmilk), the infant began to show symptoms. Another limiting factor is that this study reports the phenomenon observed in only one patient; larger sample sizers are needed to investigate this possible association.

In future studies, instrumental measures such a assessing sleep bruxism using polysomnography as well as genetical testings can be helpful as well. In addition, measuring citalopram concentration levels in the infant’s serum or her mother’s breastmilk would help make this attribution more concise. It is suggested that further studies be conducted on possible side effects and symptoms in breastfed children of mothers being treated with psychotropic medications. More studies containing larger populations are required to investigate the possible association of SSRI medications, including citalopram, with sleep bruxism. Similar results, if proven, could lead to pediatricians and other clinicians considering the possibility of medication-induced bruxism in children so that this phenomenon would not be overlooked, missed, or undiagnosed.

## Conclusion

The use of SSRI medications has been associated with symptoms of bruxism in some individuals. The case presented in this study, was a breastfed infant whose mother was using citalopram. The infant began manifesting symptoms of sleep bruxism shortly after, which resolved following the discontinuation of the medication. Similar results, if proven, could suggest that breastfed infants of mothers being treated with SSRI’s including citalopram, may present symptoms of bruxism. This could lead to a better understanding of the pathophysiological mechanisms underlying bruxism and the functions of SSRI’s as well as provide pediatricians, psychiatrists, as well as other clinicians with more holistic and effective diagnostic and treatment plans.

## Data availability statement

The raw data supporting the conclusions of this article will be made available by the authors, without undue reservation.

## Ethics statement

The studies involving human participants were reviewed and approved by the Mashhad University of Medical Sciences Research Ethics Committee. Written informed consent to participate in this study was provided by the participants’ legal guardian/next of kin. Written informed consent was obtained from the minor(s)’ legal guardian/next of kin for the publication of any potentially identifiable images or data included in this article.

## Author contributions

All authors listed have made a substantial, direct, and intellectual contribution to the work, and approved it for publication.

## Footnotes

1 Serotonin transporter (SERT or 5-HTT).
